# Supplementary material for: Low-Frequency Noise: Experiences from a Low-Frequency Noise Perceiving Population
Source: Int J Environ Res Public Health. 2023 Feb 22;20(5):3916. doi: 10.3390/ijerph20053916 (PMC10001830; doi:10.3390/ijerph20053916)
Supplement: Supplementary file 1 [file ijerph-20-03916-s001.zip › ijerph-2190268 - Supplement 1 - Tables and Figures_Low-Frequency Noise - Experiences from a Low-Frequency Noise perceiving population (1).pdf]

International Journal of
Environmental Research and Public Health

Supplements 1 – Tables and Figures

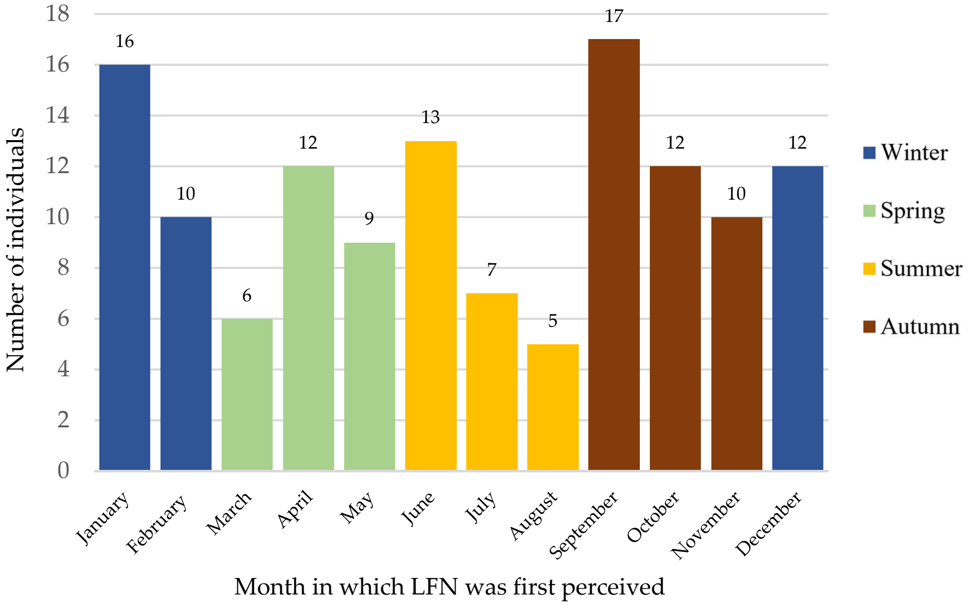

Figure S1. Month of first LNF perception. Note: Individuals reporting a month of the first LFN occurrence: n = 129.

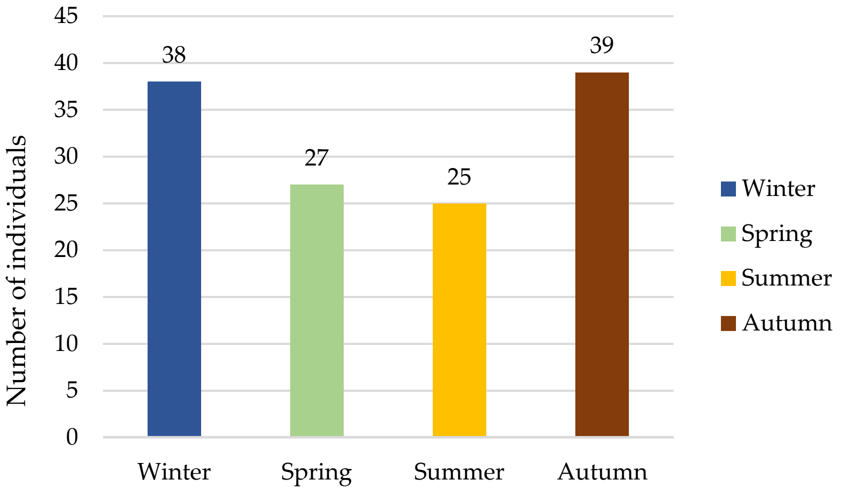

Figure S2. Season of first LNF perception. Note: Individuals reporting a season of the first LFN occurrence: n=129. The season of the first LFN was determined by the meteorological definition: Winter: December, January, February; Spring: March, April, May; Summer: June, July, August; Autumn: September, October, November.

Table S1. Type of LFN perception

**Type of perception** (multiple answers possible)	**Frequency**	**Percent total (n=190)**
Hearing	170	89.5
Feeling	153	80.5
Through the whole body	22	14.4
Specific body parts	142	92.8
Head area	111	72.6
Upper body/Torso	89	58.2
Limbs	46	30.1
Feeling in body parts touching the ground or furniture	9	5.9
Vibrations	102	53.7

Note: Underlined categories were predefined in the questionnaire.

Table S2. Type of heard LFN sound

**Type of sound** (multiple answers possible)	**Frequency**	**Percent total (n=190)**
Constant and/or non-constant sound	156	82.1
Humming	139	73.2
Droning	52	27.4
Buzzing	43	22.6
Dynamically changing sound	111	58.4
Machine sound	105	55.3
Sound similar to a thumping engine	100	52.6
Other machine sounds	7	3.6
Thumping/Throbbing	4	2.1
Pumping	3	1.6
Bass/Music	3	1.6
Pulsation	3	1.6
Other LFN types (<10%)[a]	13	6.8
Non-tonal sounds (rustling, rattling)	4	2.1
Individual answers	10	5.3

Note: Underlined categories were predefined in the questionnaire. Since multiple answers were possible, overarching categories refer to all individuals that described at least one of the underlying sounds and not the sum of the subcategories. [a] After data cleaning 30 participants provided an open "other" answer. These individuals were re-categorized into a new system depicted in this table. The "individual answers" refer to answers provided by ≤ 2 individuals.

Table S3. Number of different perceived LFN sound

Number of sounds	Frequency	Percent[a] (n=190)
1	49	25.8
2	51	26.8
3	39	20.5
4	26	13.7
5	18	9.5
6	4	2.1
7	3	1.6

Note: [a] Percent from persons reporting a type of sound, which also corresponds to the total: n=190.

Table S4. Place of felt LFN

Place of felt LFN (multiple answers possible)	Frequency[a]	Percent felt LFN[b] (n=153)	Percent total (n=190)
Through the whole body[e]	22	14.4	11.6
Body parts	142	92.8	74.7
Head area	111	72.6	58.4
<u>Pressure on ears</u>	99	64.7	52.1
Head	26	17.0	13.7
Skull	8	5.2	4.2
Ears	6	3.9	3.2
Brain	4	2.6	2.1
Individual answers	6	3.9	3.2
Upper body/Torso	89	58.2	46.8
<u>Chest</u>	71	46.4	37.4
<u>Stomach</u>	33	21.6	17.4
Back	8	5.2	4.2
Bottom/Tailbone	4	2.6	2.1
Abdomen	3	2.0	1.6
Upper body	3	2.0	1.6
Individual answers	2	1.3	1.1
Limbs	46	30.1	24.2
<u>Legs</u>	41	26.8	21.6
Feet	9	5.9	4.7
Hands	7	4.6	3.7
Arms	4	2.6	2.1
Feeling in body parts touching the ground or furniture	9	5.9	4.7

Note: Underlined categories were predefined in the questionnaire. Data was available from n=189. After data cleaning 82 participants (43.2%) provided an open "other" answer. These individuals were re-categorized into a new system depicted in this table. The "individual answers" refer to answers provided by ≤ 2 individuals. [a] Since multiple answers were possible, overarching categories refer to all individuals that described at least one of the underlying body parts and not the sum of the subcategories. [b] Percent from the number of participants that perceive LFN through feeling: n=153. d Refers to descriptions of the whole body and skin.

Table S5. Locations of LFN perception

Location		Frequency	Percent total (n=190)
Total inside		190	100
Total outside		140	73.7
Inside only		50	26.3
Inside: everywhere		11	5.8
Inside: multiple places		13	6.8
Inside and outside, but not everywhere		89	46.8
Inside everywhere	+ Outside some places	15	7.9
Inside everywhere	+ Outside around home	5	2.6
Inside multiple places	+ Outside some places	26	13.7
Inside radius around home	+ Outside radius around home	5	2.6
Inside in home	+ Outside some places	8	4.2
Inside in home	+ Outside around home	30	15.8
Inside and outside everywhere		51	26.8
Generally everywhere		32	16.8
Almost everywhere		19	10.0

Note: Underlined categories were predefined in the questionnaire. The categorization of this table was based on the pre-defined categories and open answers of specific, inside, and outside places reported.

Table S6. Number of individuals reporting a frequency associated with infrasound, LFN or above LFN frequency ranges.

Sound category	Nr. of individuals	Percent reported frequency[a]	Percent total (n=190)
Individuals providing a specific frequency	26	100	13.7
Infrasound (<20Hz)	4	15.4	2.1
LFN (20-125Hz)	21	80.8	11.1
Above LFN (125Hz-250Hz)[b]	1	3.9	0.5
Individuals reporting a frequency range/multiple frequencies	25	100	13.2
Infrasound (<20Hz)	13	52.0	6.8
LFN (20-125Hz)	23	92.0	12.1
Above LFN (125Hz-250Hz)[b]	1	4.0	0.5

Note: [a] Percent from the number of individuals who provided either a specific frequency or a frequency range/multiple ranges. Since frequencies/frequency ranges could encompass multiple categories, percentages do not add up to 100% [b] Refers to the frequency range above the definition of the Dutch Institute for Public Health and the Environment (<125Hz), but below the definitions used by other countries and/or scientists (<250Hz).

Table S7. Specific measured frequencies reported by LFN participants

LFN Frequency[a]	Nr. of individuals	Percent measured LFN (n=26)[b]	Percent total (n=190)
12	1	3.8	0.5
15	1	3.8	0.5
16	1	3.8	0.5
20	1	3.8	0.5
28	1	3.8	0.5
40	1	3.8	0.5
45	1	3.8	0.5
47	1	3.8	0.5
48	1	3.8	0.5
50	7	26.9	3.7
60	2	7.7	1.1
68	1	3.8	0.5
80	1	3.8	0.5
100	4	15.4	2.1
123	1	3.8	0.5
140	1	3.8	0.5
Total	26	100.0	13.7

Note: [a] Frequency in Hertz. [b] Percent from the number of individuals who provided a measured frequency n=26. The table does not include individuals who provided multiple frequencies or a frequency range: n=25.

Table S8. Number of different assumed LFN sources reported

Number of sources	Frequency	Percent provided assumption[a] (n=127)	Percent total (n=190)
1	62	48.8	32.6
2	28	22.0	14.7
3	16	12.6	8.4
4	12	9.4	6.3
5	6	4.7	3.2
6	1	0.8	0.5
7	1	0.8	0.5
8	0	0	0
9	1	0.8	0.5

Note: [a] Percent from the number of participants that provided an assumption: n=127.

Table S9. Assumed source of the LFN

**Assumed source** (multiple answers possible)	**Frequency**[a]	**Percent provided assumption**[b] **(n=127)**	**Percent total (n=190)**
Source unknown	62		32.6
Assumption provided	127	100.0	66.8
Air-conditioning and Ventilation	36	28.1	19.5
Mechanical ventilation	27	21.1	14.2
Air-conditioning	19	14.8	10.0
Machinery/Household appliances	33	25.8	17.4
Refrigerator/Freezer	14	10.9	7.4
Industry	11	8.6	5.8
Household appliance/washing machine	7	5.5	3.7
Heavy machinery	3	2.3	1.6
Pumps and water transport	30	23.4	15.8
Pump	12	9.4	6.3
Water pump	13	10.2	6.8
Sewage pump	8	6.3	4.2
Electrical installations	29	22.7	15.3
Electricity station/transfer house	10	7.8	5.3
Other electrical installations	10	7.8	5.3
Generator	8	6.3	4.2
Meter box	3	2.3	1.6
Traffic[c]	25	19.5	13.2
Heating	22	17.2	11.6
Central heating system	14	10.9	7.4
District heating	5	3.9	2.6
Underfloor heating	3	2.3	1.6
Gas extraction and transport	19	14.8	10.0
Gas transport	15	11.7	7.9
Gas extraction	5	3.9	2.6
Other assumed sources (<10%)[d]	45	35.2	23.6
Wind farm	15	11.7	7.9
Audio-video/nearby music	9	7.0	4.7
Natural sources/weather	6	4.7	3.2
Wireless services	6	4.7	3.2
Construction	3	2.3	1.6
Radiation/electromagnetic fields	5	3.9	2.6
Individual answers	3	2.3	1.6

Note: Underlined categories were predefined in the questionnaire. Data was available from n=189. [a] Since multiple answers were possible, overarching categories refer to all individuals that described at least one of the underlying sources and not the sum of the subcategories. [b] Percent from the number of participants that provided an assumption: n=127. [c] All traffic including cars, trains, boats, metro, and aircraft. [d] After data cleaning 91 participants (47.9%) provided an open “other” answer. These individuals were re-categorized into a new system depicted in this table. The “individual answers” refer to answers provided by ≤ 2 individuals. The predefined categories of “wind farm” and “audio-video” were selected by less than 10% of the participants and were counted in the overarching “other” category.

Table S10. Circumstances influencing the LFN perception

**Circumstance** (multiple answers possible)		**Frequency**[a]	**Percent circumstance**[b]	**Percent total (n=190)**
LFN always perceived the same way		45		23.7
LFN depends on a circumstance		144		75.8
Time of the day[c]		81	100	42.6
**Morning**		**36**	**44.4**	**19.0**
Time Specification		19	23.5	10.0
No Time Specification		17	21.0	9.0
**Afternoon**		**16**	**20.0**	**8.4**
Time Specification		4	4.9	2.1
No Time Specification		12	14.8	6.3
**Evening**		**42**	**51.9**	**22.1**
Time Specification		16	19.8	8.4
No Time Specification		26	32.1	13.7
**Night**		**66**	**81.5**	**34.7**
Time Specification		21	25.9	11.1
No Time Specification		45	55.6	23.7
Presence of other sounds		76	100	40.0
Masking effect		59	77.6	31.1
General/ambient noise		43	56.6	22.6
Traffic		11	14.5	5.8
Storm/Wind/Rain		8	10.5	4.2
Music/Radio		8	10.5	4.2
TV		7	9.2	3.7
Other specific sounds		9	11.8	4.7
Amplification of noise		3	4.0	1.6
Wind		30	100	15.8
Wind direction		14	46.7	7.4
Wind presence/strength		13	43.3	6.8
Stronger wind, less LFN		6	20.0	3.2
Stronger wind, more LFN		4	13.3	2.1
Type of influence unclear		2	6.7	1.1
Season		28	100	14.7
Winter	LFN Worse/More	12	42.9	6.3
	LFN Better/Less	1	3.6	.5
Spring	LFN Worse/More	4	14.3	2.1
	LFN Better/Less	0	0	0
Summer	LFN Worse/More	4	14.3	2.1
	LFN Better/Less	4	14.3	2.1
Autumn	LFN Worse/More	6	21.4	3.2
	LFN Better/Less	0	0	0

Day of the week	28	100	14.7
Frequency of days where LFN is worse/present:			
One/two days per week	8	28.6	4.2
Many days per week	7	25.0	3.7
Almost all days per week	8	28.6	4.2
LFN differing on weekends/holidays:	19	67.8	10.0
LFN Worse/More	12	42.9	6.3
LFN Better/Less	7	25.0	3.7
Changing without a system	33	100	17.4
Other[d]	53	100	27.9
Whether	16	30.2	8.4
Humidity/Rain	12	22.6	6.3
More humidity/Rain - LFN worse/more	9	17.0	4.7
Effect not described	3	5.7	1.6
Temperature	15	28.3	7.9
Warmth LFN worse/more	7	13.2	3.7
Cold/frost LFN worse/more	6	11.3	3.2
LFN Better/Less	2	3.8	1.1
Depends on a suspected LFN source being turned on	14	26.4	7.4
Distraction	6	11.3	3.2
Stress dependent	3	5.7	1.6
Individual answers	9	17.0	4.7

Note: Underlined categories were predefined in the questionnaire. [a] Since multiple answers were possible, overarching categories refer to all individuals that described at least one of the underlying circumstances and not the sum of the subcategories. [b] Percent from the corresponding overarching category. [c] Time periods refer to the following hours: Morning: 6.00 – 12.00, Afternoon: 12.00 – 18.00, Evening: 18:00 – 24.00, Night: 24.00 – 6.00. [d] After data cleaning 73 participants (38.4 %) provided an open “other” answer. These individuals were re-categorized into a new system depicted in this table. The “individual answers” refer to answers provided by ≤ 2 individuals. The predefined category of “temperature” was selected by less than 10% of the participants and was counted in the overarching “other” category.

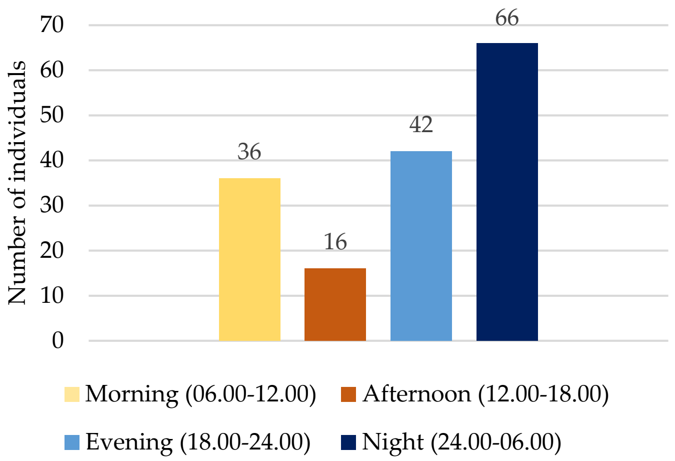

Figure S3. LFN perception depending on the time of the day. Note: Individuals reporting their LFN perception to depend on the time of the day: n=81.

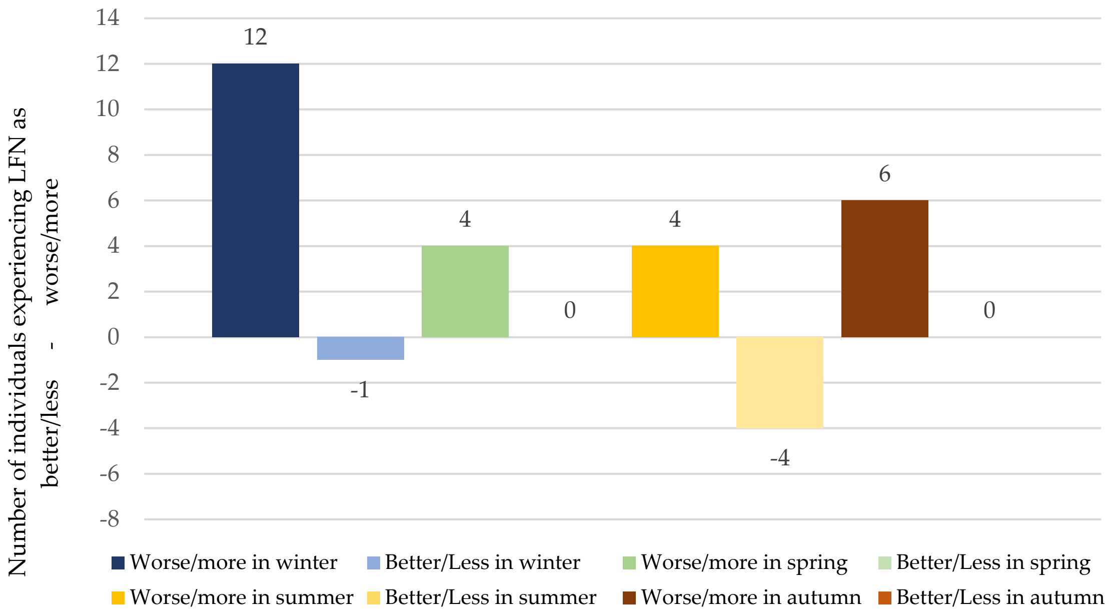

Figure S4. LFN perception depending on season. Note: Individuals reporting their LFN perception to depend on the season: n=28.

Table S11. Number of other persons perceiving the LFN

Number of persons	Frequency	Percent other person[a] (n=128)	Percent total (n=190)
1	62	48.4	32.6
2	39	30.5	20.5
3	20	15.6	10.5
4	4	3.1	2.1
5	2	1.6	1.1

Note: [a] Percent from the number of participants that reported other persons to also perceive the LFN: n=128.

Table S12. Other persons perceiving the LFN

**Person** (multiple answers possible)	**Frequency**	**Percent other person[a] (n=128)**	**Percent total (n=190)**
No other person	61		32.1
Other persons	128	100.0	67.4
Partner	53	41.4	27.9
Guest	38	29.7	20.0
Neighbor	37	28.9	19.5
Family member	35	27.3	18.4
Other[b]	57	44.5	30.0
Housemates	13	10.2	6.8
Friends	10	7.8	5.3
Locals	10	7.8	5.3
Researchers/Measurement employees	7	5.5	3.7
Colleagues	7	5.5	3.7
Acquaintances/Individual answers	17	13.3	9.0

Note: Underlined categories were predefined in the questionnaire. Data was available from n=189. Since multiple answers were possible, the percentages do not represent the sum of the categories. [a] Percent from the number of participants that reported other persons to also perceive the LFN: n=128. [b] After data cleaning 40 participants (21.1 %) provided an open "other" answer. These individuals were re-categorized into a new system depicted in this table. The "Individual answers" refer to answers provided by ≤ 2 individuals. The predefined categories of "Housemates" and "Colleagues" were selected by less than 10% of the participants and were counted in the overarching "other" category.

Table S13. Number of named actions to reduce nuisance from LFN

**Number of actions**	**Frequency**	**Percent named actions[a] (n=183)**	**Percent total (n=190)**
1	10	5.5	5.3
2	12	6.6	6.3
3	24	13.1	12.6
4	40	21.9	21.1
5	54	29.5	28.4
6	27	14.8	14.2
7	11	6.0	5.8
8	2	1.1	1.1
9	2	1.1	1.1
15	1	0.5	0.5

Note: [a] Percent from the number of participants that reported to have tried actions to reduce nuisance from LFN: n=183.

Table S14. Actions to reduce nuisance due to LFN and their success

**Action** (multiple answers possible)	**Frequency**[a]	**Percent**[b] **(n=190)**	**Reduction of annoyance**			
			**Yes**	**(%)**[c]	**No**	**(%)**[c]
No actions taken	6	3.2				
Actions taken	183	96.3				
Noise reduction	165	86.8				
Putting in earplugs/earphones	163	85.8	26	16.0	124	76.1
Noise-cancelling headphones	7	3.7	6	85.7	1	14.3
Placing something other than earplugs in or on ears	7	3.7	6	85.7	1	14.3
Masking sound	155	81.6				
Turning up/louder radio or television	145	76.3	78	53.8	59	40.7
White noise	10	5.3	6	60.0	2	20.0
Ventilation	10	5.3	7	70.0	1	10.0
Other noise	9	4.7	5	55.5	1	11.1
Music via headphones	5	2.6	3	60.0	1	20.0
Turning on music	3	1.6	2	66.7	0	0
Headphones	3	1.6	1	33.3	0	0
Turning off a suspected source/ Switching off electricity/battery/equipment[c]	143	75.3	18	12.6	123	86.0
Changing/adapting living location	143	75.3				
Closing or opening windows	142	74.7	28	19.6	103	72.0
Insulation of walls or windows	6	3.2	3	50.0	0	0
Being at a different location	6	3.2	3	50.0	2	33.3
Changing/adapting sleep location	108	56.8				
Changing position of bed	107	56.3	14	13.0	89	83.2
Sleeping at different location	9	4.7	1	11.1	3	33.3
Placing bed on dampening material	5	2.6	3	60.0	1	20.0
Change of mattress	4	2.1	2	50.0	2	50.0
Other[d]	35					
Contacting authorities/neighbors	8	4.2	1	12.5	4	50.0
Distraction	5	2.6	4	80.0	0	0
Coping/positive thoughts/coaching	4	2.1	4	100.0	0	0
Searching for source	4	2.1	0	0	2	50.0
Medication/substances	3	1.6	3	100.0	0	0
Individual answers	11	5.8	5	45.4	2	18.2

Note: Underlined categories were predefined in the questionnaire. Data was available from n=189.[a] Since multiple answers were possible, overarching categories refer to all individuals that described at least one of the underlying actions and not the sum of the subcategories. [b] Percent from the corresponding overarching category. Percentages do not add up due to missing responses regarding the success of annoyance reduction or due to unclear responses stating that an action was partly useful, partly not. [c] The predefined answer options "Turning off a suspected source" and "Switching off electricity/battery /equipment" were combined into one category due to the considerable overlap between the two categories. All participants were combined in this question that chose at least one of the two answer options. [d] After data cleaning 84 participants (44.2%) provided an open "other" answer. These individuals were re-categorized into a new system depicted in this table. The "Individual answers" refer to answers provided by ≤ 2 individuals.

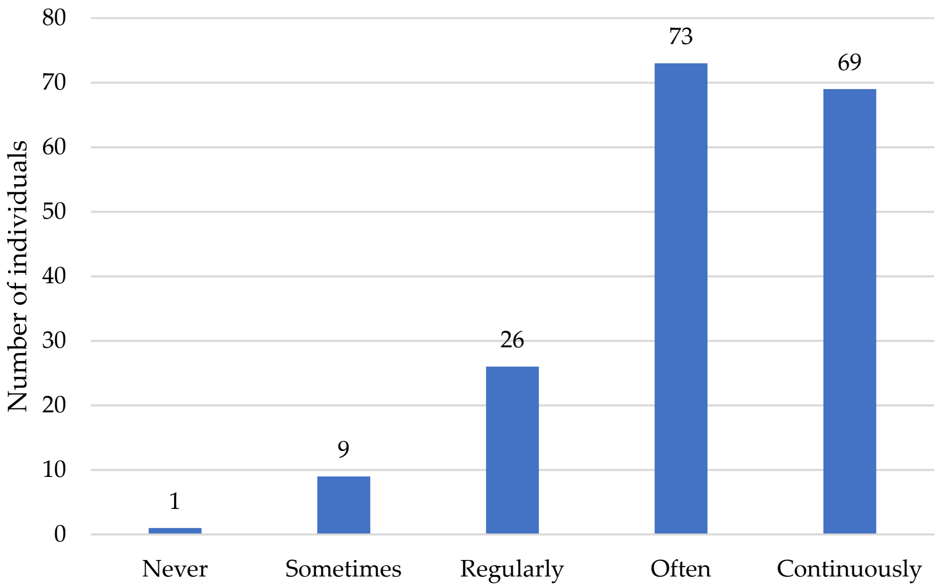

Figure S5. Frequency of LFN-related experienced complaints as indicated by the LFN group. Note: LFN group n=190. Data available from n=178.

Table S15. Number of physical and psychological complaints

Number of complaints	Frequency	Percent reported complaint[a] (n=187)	Percent total (n=190)
1	4	2.1	2.1
2	6	3.2	3.2
3	10	5.3	5.3
4	8	4.3	4.2
5	13	7.0	6.8
6	14	7.5	7.4
7	17	9.1	8.9
8	13	7.0	6.8
9	16	8.6	8.4
10	19	10.2	10.0
11	14	7.5	7.4
12	10	5.3	5.3
13	9	4.8	4.7
14	9	4.8	4.7
15	6	3.2	3.2
16	6	3.2	3.2
17	1	.5	.5
18	3	1.6	1.6
19	5	2.7	2.6
20	1	.5	.5
21	2	1.1	1.1
30	1	0.5	0.5

Note: [a] Percent from the number of participants that reported at least one physical or psychological complaint: n=187.

Table S16. Number of social and societal consequences

Number of actions	Frequency	Percent reported consequence[a] (n=114)	Percent total (n=190)
1	68	59.6	35.8
2	29	25.4	15.3
3	9	7.9	4.7
4	5	4.4	2.6
5	3	2.6	1.6

Note: [a] Percent from the number of participants that reported at least one social or societal problem: n=114.

Table S17. Number of different authorities LFN was reported to

Number of authorities	Frequency	Percent authority report[a] (n=174)	Percent total (n=190)
1	35	20.1	18.4
2	39	22.4	20.5
3	41	23.6	21.6
4	37	21.3	19.5
5	12	6.9	6.3
6	4	2.3	2.1
7	3	1.7	1.6
8	2	1.1	1.1
9	1	0.6	0.5

Note: [a] Percent from the number of participants that reported LFN to an authority: n=174.

Table S18. Authorities LFN was reported to

Authority (multiple answers possible)	Frequency	Percent authority report[a] (n=174)	Percent total (n=190)
No authority contacted	14		7.4
Authority contacted	174	100.0	91.6
Municipality	132	75.9	69.5
Dutch Municipal Health Service (GGD)	108	62.1	56.8
Provincial Environmental Service[b]	85	48.9	44.7
Low-Frequency noise foundation or other noise/LFN related interest groups	41	23.6	21.6
Institute for Public Health and the Environment (RIVM)	38	21.8	20.0
Housing association	37	21.3	19.5
Companies/owners of assumed source of sound	19	10.9	10.0
Other[c]	42	24.1	22.1
Environmental agency[d]	14	8.1	7.4
Police	10	5.8	5.3
Media	5	2.9	2.6
Politics	4	2.3	2.1
Personal environment	4	2.3	1.6
Owners association	3	1.7	1.6
Communal association	3	1.7	1.6
Legal assistance	2	1.2	1.1
Individual answers	6	3.5	3.2

Note: Underlined categories were predefined in the questionnaire. Since multiple answers were possible, the percentages do not represent the sum of the categories. [a] Percent from the number of participants that reported LFN to an authority: n=175. [b] Referring to the Dutch "provinciale omgevingsdienst". [c] After data cleaning 89 participants (46.8%) provided an open "other" answer. These individuals were re-categorized into a new system depicted in this table. The "Individual answers" refer to answers provided by ≤ 2 individuals. [d] Referring to the Dutch "milieudienst".

Table S19. Number of experts consulted concerning experienced complaints

Number of experts	Frequency	Percent expert report[a] (n=152)	Percent total (n=190)
1	53	34.9	27.9
2	33	21.7	17.4
3	30	19.7	15.8
4	16	10.5	8.4
5	8	5.3	4.2
6	7	4.6	3.7
7	3	2.0	1.6
8	1	0.7	0.5
9	1	0.7	0.5

Note: [a] Percent from the number of participants that reported to have consulted an expert: n=152

Table S20. Experts consulted concerning experienced complaints

Expert (multiple answers possible)	Frequency	Percent expert report[a] (n=152)	Percent total (n=190)
No expert consulted	38		20.0
Expert consulted	152	100.0	80.0
General practitioner	130	85.5	68.4
Audiologist	68	44.7	35.8
ENT physician	61	40.1	32.1
Psychologist	41	27.0	21.6
Neurologist	20	13.2	10.5
Other[b]	54	35.5	28.4
Social work	16	10.5	8.4
Psychiatrist	12	7.9	6.3
Cardiologist	9	5.9	4.7
Hearing nonrelated specialist doctor[c]	8	5.3	4.2
Hearing/acoustic related expert	7	4.6	3.7
Experts for other forms of medicine/therapy	4	2.6	2.1
Sound measuring expert	2	1.3	1.1
Individual answers	11	7.2	5.8

Note: Underlined categories were predefined in the questionnaire. Since multiple answers were possible, the percentages do not represent the sum of the categories. [a] Percent from the number of participants that reported to have consulted an expert: n=152. [b] After data cleaning 30 participants (15.8%) provided an open "other" answer. These individuals were re-categorized into a new system depicted in this table. The remaining "other" section refers to answers that were provided by two or less individuals. The "Individual answers" refer to answers provided by ≤ 2 individuals. [c] This included the experts: dental specialist, eye doctor, gynecologist, skin doctor and medical examiner.

Table S21. Medication usage of the LFN and comparison group

**Medication type (multiple answers possible)**	**LFN (n=190)**	**CG (n=371)**	χ^2	*df*	*p*	*V*
Taking any medication (%[a])	109 (57.4)	156 (42.0)	11.83	1	**<.001*****	.15
Calming medication (%)	44 (23.2)	11 (3.0)	57.90	1	**<.001*****	.32
Cardiovascular medication (%)	44 (23.2)	88 (23.7)	0.02	1	.88	<.01
Other medication (%)	76 (40.0)	99 (26.7)	10.38	1	**<.001*****	-.14
	**Mean ± SD (Range)**		***U***		***p***	***r***
Nr. of different medications taken[a]	2.59±1.75 (1-10)	2.03±1.50 (1-8)	6687.0		**.003****	-.18
Nr. of types of medication[b]	1.92 ± 1.20 (1-6)	1.56 ± .94 (1-6)	6726.0		**.005****	-.18

Note: Medication referred to also prescription-free medication, but excluded vitamins, food supplements, anticonception or alternative forms of therapy. Named medications were categorized into calming medication (including i.e. sleep medication and antidepressant/antipsychotic medication), cardiovascular medication and other medication (including i.e.: skin, respiratory, pain, or gastrointestinal medication). [a] Based on: LFN=109, CG=263. [b] Based on: LFN=109, CG=260. Possible type of medications were calming, cardiovascular or subcategories of other medication (including i.e.: skin, respiratory, pain, or gastrointestinal medication). LFN = LFN group. CG = comparison group. ** significant difference at a level p < .01. *** significant difference at a level p < .001.

Table S22. Number of different medications taken by individuals of the LFN and comparison group

**Number of medications**	**LFN**			**CG**		
	**Frequency**	**Percent reported medication[a] (n=109)**	**Percent total (n=190)**	**Frequency**	**Percent reported medication[a] (n=154)**	**Percent total (n=371)**
1	39	35.8	20.5	79	51.3	21.3
2	23	21.1	12.1	38	24.7	10.2
3	18	16.5	9.5	13	8.4	3.5
4	16	14.7	8.4	10	6.5	2.7
5	7	6.4	3.7	9	5.8	2.4
6	3	2.8	1.6	2	1.3	0.5
7	1	0.9	0.5	2	1.3	0.5
8	0	0	0	1	0.6	0.3
9	1	0.9	0.5	0	0	0
10	1	0.9	0.5	0	0	0

Note: [a] Percent from the number of participants that reported to take medication: LFN=109, CG=154.

Table S23. Number of types of medication taken by individuals of the LFN and comparison group

Number of medication types	LFN			CG		
	Frequency	Percent reported medication types[a] (n=109)	Percent total (n=190)	Frequency	Percent reported medication types[a] (n=220)	Percent total (n=371)
1	52	47.7	27.4	99	65.6	26.7
2	34	31.2	17.9	30	19.9	8.1
3	11	10.1	5.8	16	10.6	4.3
4	6	5.5	3.2	3	2.0	0.8
5	4	3.7	2.1	2	1.3	0.5
6	2	1.8	1.1	1	0.7	0.3

Note: [a] Percent from the number of participants that reported to take medication that could be categorized into a medication type: LFN=109, CG=220.

Table S24. Current occupation in the LFN group, comparison group, and the Dutch population

Current occupation	LFN (n=187)	CG (n=364)	DP[a] (n=17,475)	Comparison LFN and CG			
				χ^2	*df*	*p*	*φ*
No current occupation (%[b])	58 (30.5)	159 (42.9)	8,837 (50.6)	7.71	1	**.005****	-.12
Having a current occupation	130 (68.4)	211 (56.9)	8,638 (49.4)				

Note: Group comparisons were conducted between the LFN and CG group. No current occupations refers to persons not eligible for work or who´s daily living status is best described with something else than work i.e. pension, students, or homemaker. Current occupation refers to persons currently working or work eligible individuals who would be usually working i.e. individuals currently incapacitated, on sick leave or unemployed. [a] Data from the CBS from the fourth quarter of 2020. The number of individuals is provided in 1,000 steps. [b] Percentages refer to the total of the groups: LFN=190, CG=371, DP=17,475,415 as of 1 January 2021. Data was available from LFN0187, CG0364. LFN = LFN group, CG = Comparison group, DP = Dutch population. ** significant difference at a level p ≤ 0.01.

Table S25. Field of occupation in the LFN group, comparison group, and the Dutch population

	LFN (n=130)	CG (n=211)	DP[a] (n=8,638)	Comparison LFN and CG			
				χ^2	*df*	*p*	*φ* *V*[b]
Educational professions(%[c])	14 (10.8)	30 (14.2)	603 (7.0)	.84	1	.36	-.05
Creative and linguistic professions	10 (7.7)	9 (4.3)	216 (2.5)	1.15	1	.28	.06
Commercial professions	11 (8.5)	10 (4.7)	918 (10.6)	1.95	1	.16	.08
Business and administrative professions	20 (15.4)	56 (26.5)	1,661 (19.2)	5.78	1	**.016***	-.13
Managers	13 (10.0)	25 (11.8)	393 (4.5)	0.56	1	.45	-.04
Public administration, security and jurisdiction.	3 (2.3)	7 (3.3)	319 (3.7)			.75	.03
Technical occupations	21 (16.2)	17 (8.1)	1,191 (13.8)	4.52	1	**.034***	.12
ICT professions	6 (4.6)	6 (2.8)	429 (5.0)			.38	.05
Agricultural professions	1 (0.8)	2 (1.0)	185 (2.1)			1.00	.01
Care and welfare professions	33 (25.4)	34 (16.1)	1,301 (15.1)	3.80	1	.05	.11
Service professions	8 (6.2)	6 (2.8)	714 (8.3)	1.43	1	.23	.07
Transport and logistics professions	3 (2.3)	8 (3.7)	572 (6.6)			.54	.04
Other	-	-	133 (1.5)				

Note: Group comparisons were conducted between the LFN and CG group. Multiple jobs were reported by LFN=18 and CG=5, from which 12 (LFN) and 5 (CG) fell into 2 different occupational categories. In these cases, both categories were counted. Due to these multiple answers, the percentages do not represent the sum of all categories. [a] Data from the CBS from the fourth quarter of 2020. The number of individuals is provided in 1,000 steps. [b] Results are based on Fisher's exact test and Cramer's V for the categories Public administration, Security and jurisdiction, ICT professions, Agricultural professions, and Transport and logistics. [c] Percentages refer to the number of participants that have a current occupation: LFN=130, CG=211, DP=8,638,000 as of 1 January 2021. LFN = LFN group, CG = Comparison group, DP = Dutch population. * significant difference at a level p < .05.

Table S26. Province distribution of the LFN group, comparison group and Dutch population living in the Netherlands

Province (%)	LFN (n=188)	CG (n=364)	DP[a] (n=17.475)	Comparison LFN and CG			
				χ^2	*df*	*p*	*φ*
Drenthe	8 (4.3)	21 (5.8)	495 (2.8)	0.57	1	.45	.03
Flevoland	3 (1.6)	13 (3.6)	428 (2.5)	1.72	1	.19	.06
Friesland	10 (5.3)	10 (2.7)	651 (3.7)	2.35	1	.13	-.07
Gelderland	26 (13.8)	34 (9.3)	2,097 (12.0)	2.58	1	.22	-.07
Groningen	18 (9.6)	11 (3.0)	587 (3.4)	10.69	1	**.001*****	-.14
Limburg	3 (1.6)	31 (8.5)	1,116 (6.6)	10.27	1	**.001*****	.14
Noord-Brabant	27 (14.4)	59 (16.2)	2,574 (14.7)	.32	1	.57	.03
Noord-Holland	39 (20.7)	49 (13.5)	2,889 (16.5)	4.91	1	**.027***	-.09
Overijssel	9 (4.8)	18 (4.9)	1,167 (6.7)	0.07	1	.94	<-.01
Utrecht	13 (6.9)	24 (6.6)	1,361 (7.8)	0.02	1	.89	<-.01
Zeeland	6 (3.2)	12 (3.3)	385 (2.2)	<0.01	1	.95	<-.01
Zuid-Holland	26 (13.9)	82 (22.5)	3,726 (21.3)	5.96	1	**.015***	.10

Note: Group comparisons were conducted between the LFN and CG group. [a] Data from the CBS from 1 January 2021. The number of individuals is provided in 1,000 steps. Percentages refer to the total number of participants living in the Netherlands: LFN=188, CG:364, DP=17,475,415 as of 1 January 2021. LFN = LFN group, CG = Comparison group, DP = Dutch population.* significant difference at a level $p < .05$. *** significant difference at a level $p \leq .001$.

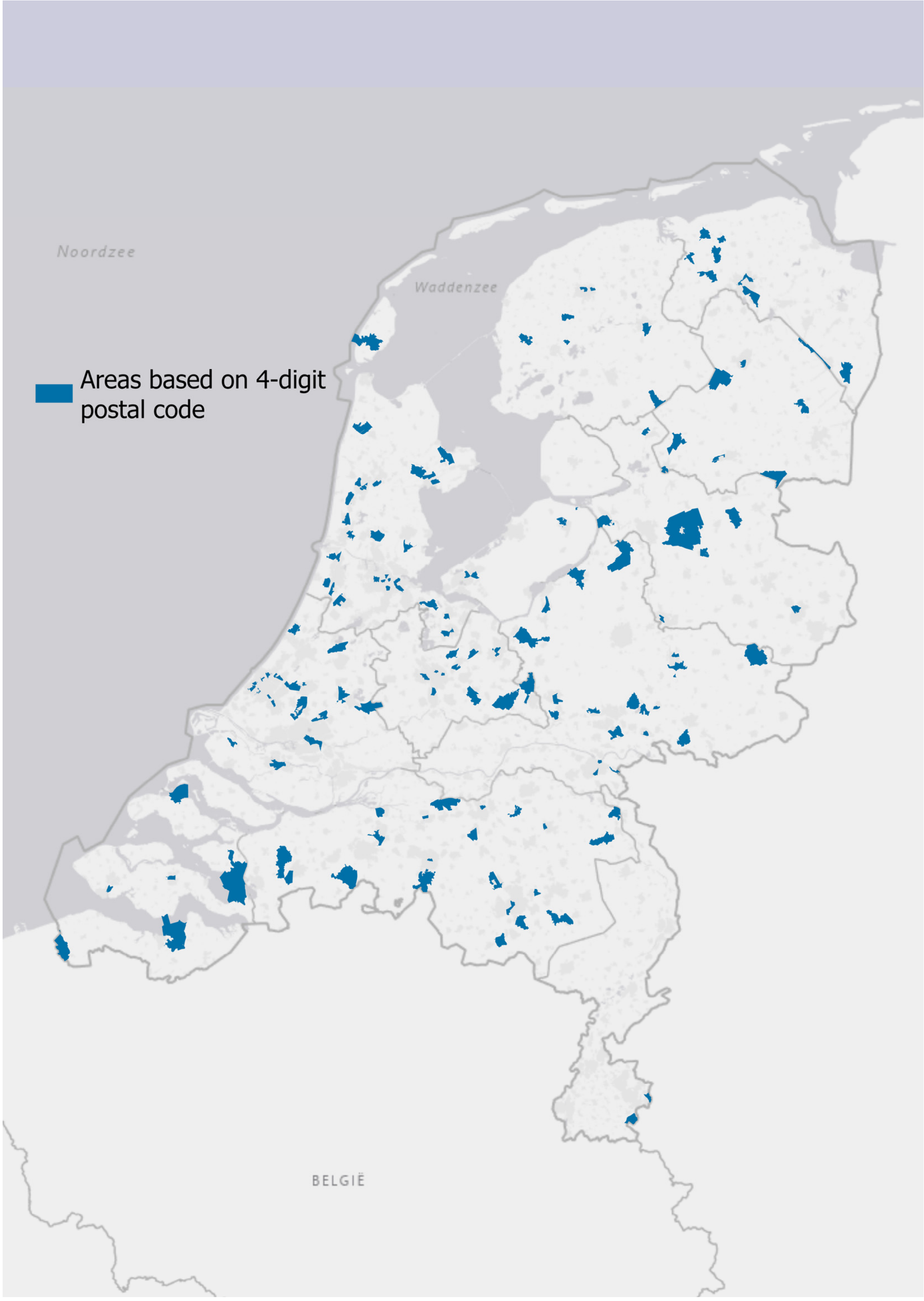

Figure S6. Geographical distribution of LFN participants` living place based on the Dutch four-digit postal codes. Note: Data was available from all participants living in the Netherlands: n=188. In total, 173 different postal code areas are depicted; 12 postal codes entail two participants and one postal code three participants. The size of postal codes does not relate to the area where noise-complaints are perceived, since postal code areas vary substantially and are typically smaller in urban areas compared to rural areas.

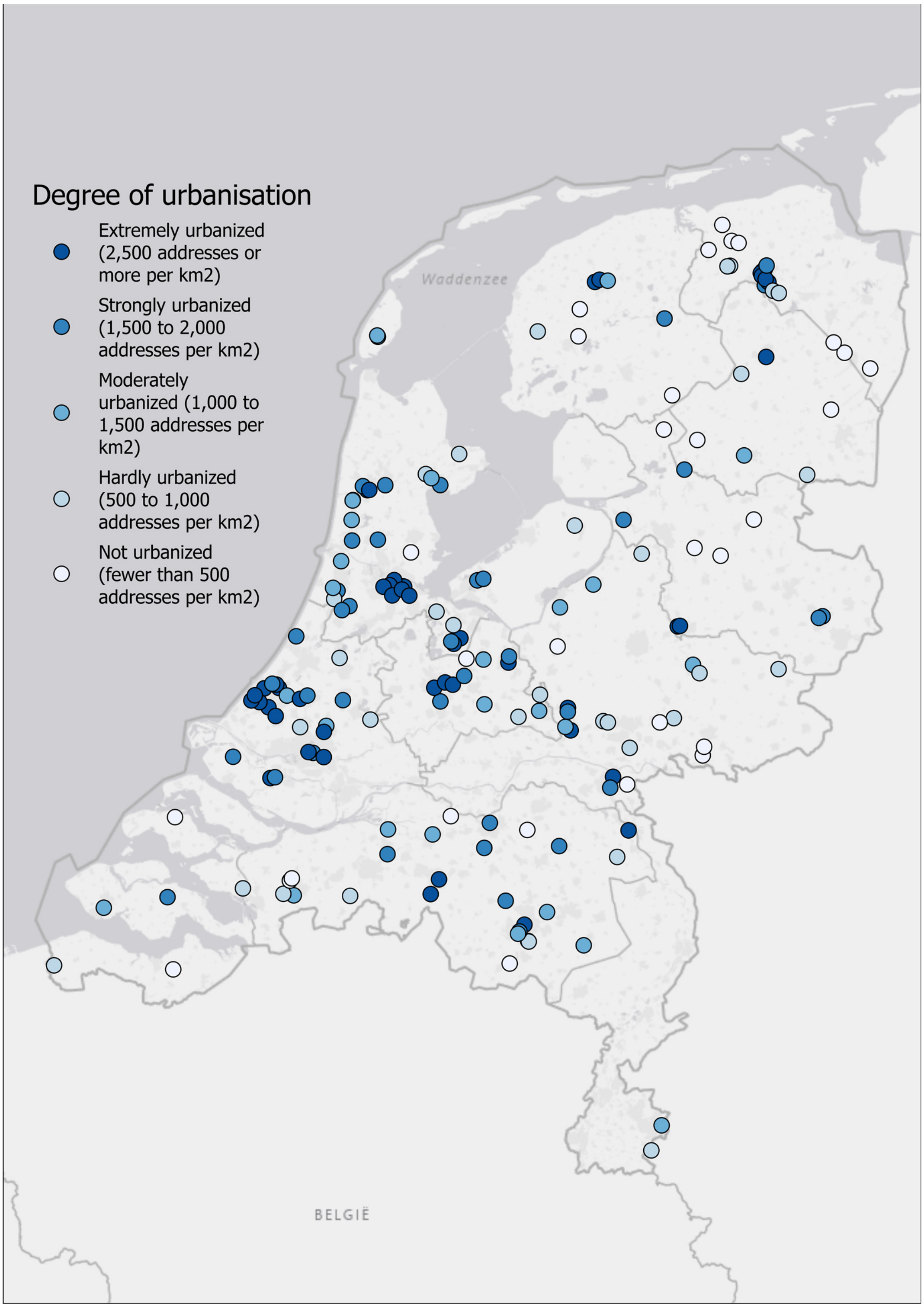

Figure S7. Geographical distribution of the urbanization level of the LFN participants` living location in the Netherlands. Note: Data was available from all participants living in the Netherlands: n=188.

Table S27. Urbanization level of the living location of the LFN group and Dutch population

Urbanization level[a]	LFN (n=188)		DP (n=17.475)	
	**Frequency**	**Percent[b]**	**Frequency[c]**	**Percent[b]**
Extremely urbanized	52	27.4	4,227	24.2
Strongly urbanized	39	20.5	4,384	25.1
Moderately urbanized	29	15.3	2,960	16.9
Hardly urbanized	34	17.9	2,958	16.9
Not urbanized	34	17.9	2,947	16.9

Note: [a] Urbanization levels are defined as: Extremely urbanized - 2,500 addresses or more per km2; Strongly urbanised: 1,500 to 2,000 addresses per km2; Moderately urbanised: 1,000 to 1,500 addresses per km2; Hardly urbanised: 500 to 1,000 addresses per km2; Not urbanised: fewer than 500 addresses per km2. [b] Percentages refer to the total number of participants living in the Netherlands: LFN=188, DP=17,475,415 as of 1 January 2021. [c] Data from CBS from January 1st 2021. The number of individuals is provided in 1,000 steps. LFN = LFN group, DP = Dutch population.

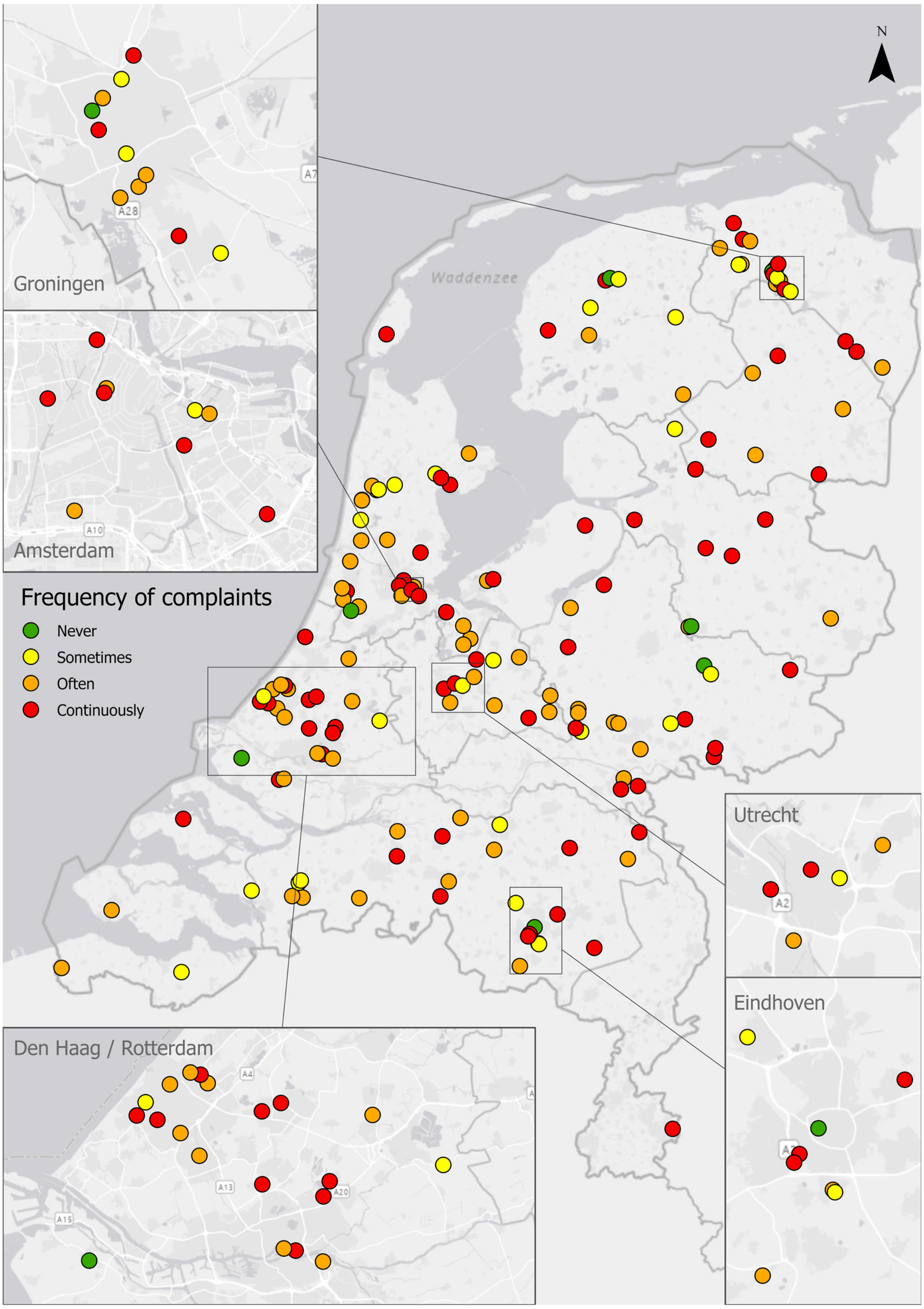

Figure S8. Geographical distribution of the frequency of LFN complaints based on the LFN participants` living location in the Netherlands and main urban regions. Note: Data was available from: n=178.

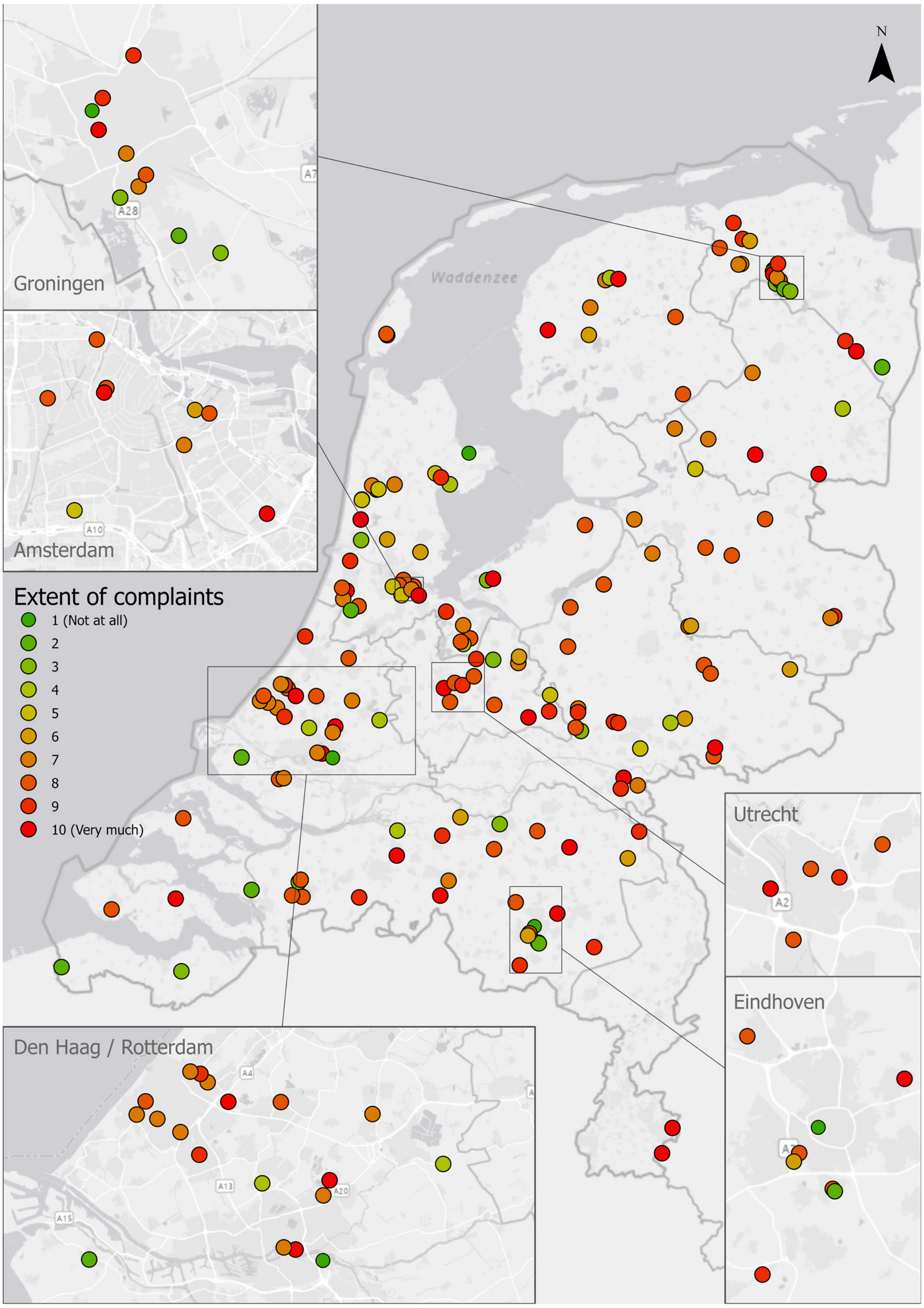

Figure S9. Geographical distribution of the extent of experienced LFN complaints based on the LFN participants` living location in the Netherlands and main urban regions. Note: Data was available from: n=183.
