# Supplementary material for: Low-Frequency Noise: Experiences from a Low-Frequency Noise Perceiving Population
Source: Int J Environ Res Public Health. 2023 Feb 22;20(5):3916. doi: 10.3390/ijerph20053916 (PMC10001830; doi:10.3390/ijerph20053916)
Supplement: Supplementary file 1 [file ijerph-20-03916-s001.zip › ijerph-2190268 - Supplement 2 - Administered questionnaire_Low-Frequency Noise - Experiences from a Low Frequency Noise perceiving population.pdf]

International Journal of
Environmental Research and Public Health

MDPI

Supplements 2 – Administered questionnaire

Persoonlijke gegevens

Wat is uw leeftijd (in jaren)?

☐

Wat is uw geslacht?

☐ Man

☐ Vrouw

Waar woont u?

Land

Provincie

Plaats

Postcode cijfers

Schoolopleiding

☐ Lagere school

☐ LBO, VMBO basis, VMBO kader, VMBO-gl, LTS of LEAO

☐ MBO, VMBO-t, MULO, MAVO, MTS of MEAO

☐ HAVO

☐ Atheneum / Gymnasium

☐ HBO,HEAO,PabooofHTS

☐ Universiteit – Bachelor

☐ Universiteit – Master

☐ Anders, namelijk

Hoeveel jaren heeft u onderwijs gevolgd (het gaat om alle jaren onderwijs vanaf de basisschool (of lagere school) en inclusief de middelbare school en alle eventuele vervolgopleidingen)?

Wat is uw burgerlijke staat?

☐ Gehuwd

☐ Samenwonend

☐ Partner, niet samenwonend

☐ Ongehuwd

☐ Gescheiden

☐ Weduwe / wedunaar

☐ Anders, namelijk

Hoe ziet uw huishouden eruit?

☐ Alleenwonend.

☐ Samenwonend met andere mensen.

Aantal mensen waarmee u samenwoont:

Wat doet u in het dagelijks leven (meerdere antwoordopties mogelijk)?

☐ Ik werk full-time

Hoeveel uren per week werkt u? ________________

☐ Ik werk part-time

Hoeveel uren per week werkt u? ________________

☐ Ik studeer

☐ Ik ben huisvrouw, huisman

☐ Ik ben arbeidsongeschikt

Voor hoeveel % bent u arbeidsongeschikt?__________________%

☐ Ik ben met pensioen of prepensioen

☐ Ik ben werkloos

Hoe lang bent u al werkloos? ___________________

☐ Ik ben met ziekteverlof.

Voor hoeveel % bent u ziekgemeld?___________________%

☐ Ik doe iets anders, namelijk _______________________

International Journal of
Environmental Research
and Public Health

MDPI

Wat is uw beroep? Of waar bent u voor opgeleid?

In wat voor soort woning woont u?

- ☐ Flat of etagewoning
- ☐ Tussenwoning in een rij
- ☐ Hoekwoning in een rij
- ☐ Twee onder één kap
- ☐ Vrijstaand

Hoe lang woont u al in deze woning (in jaren)?

Vragen met betrekking tot het waargenomen geluid

Wanner hebt u het geluid voor het eerst waargenomen (jaartal)?

Wat voor soort geluid neemt u waar?

- ☐ Bromtoon
- ☐ Gonzend geluid
- ☐ Zoemend geluid
- ☐ Geluid dat lijkt op dreunende dieselmotor
- ☐ Trillingen
- ☐ Anders:

Wat is naar uw mening de oorzaak/de bron van het geluid?

- ☐ Ik weet het niet
- ☐ Air-conditioning
- ☐ Koelkast/Diepvries
- ☐ Aquarium/Terrarium/Binnenvijver
- ☐ C.v.-installatie
- ☐ Vloerverwarming
- ☐ Stadsverwarming
- ☐ Mechanische ventilatie
- ☐ Meterkast
- ☐ Schakelklokken
- ☐ Audio-video
- ☐ Windpark
- ☐ Verkeer (auto's, treinen, boten, vliegtuigen)
- ☐ Anders:

Is er onderzoek gedaan naar het laag frequentgeluid of heeft u dit aangevraagd?

☐ Ja

☐ Nee

Zo ja, kon het laag frequentegeluid worden waargenomen/gemeten?

☐ Ja

☐ Nee

Zo ja, wat was de frequentie van het laagfrequente geluid in Hertz?

Waar neemt u het geluid waar?

☐ Overal

☐ Op specifieke plaatsen (geef a.u.b. hieronder een beschrijving):

☐ Binnen

- ☐ Overal binnen
- ☐ Alleen in bepaalde kamers/locaties (geef a.u.b. hieronder een beschrijving):

☐ Buiten

- ☐ Overal buiten
- ☐ Alleen op bepaalde locaties (geef a.u.b. hieronder een beschrijving):

Hoe neemt u het geluid waar?

- ☐ Horen
- ☐ Druk op de oren
- ☐ Voelen
  - ☐ Borst
  - ☐ Maag
  - ☐ Benen
  - ☐ Andere lichaamsdelen (geef a.u.b. hieronder een beschrijving)
- ☐ Anders:

Hangt uw waarneming van het laagfrequente geluid af van specifieke situaties (meerdere antwoordopties mogelijk)?

- ☐ Nee, ik neem het geluid altijd op dezelfde manier waar.
- ☐ Van het jaargetijde. Geef a.u.b. hieronder een beschrijving:
- ☐ Van de dag van de week. Geef a.u.b. hieronder een beschrijving:
- ☐ Van het tijdstip. Geef a.u.b. hieronder een beschrijving:
- ☐ Van de aanwezigheid van andere geluiden. Geef a.u.b. hieronder een beschrijving:
- ☐ Van windrichting. Geef a.u.b. hieronder een beschrijving:
- ☐ Van de temperatuur. Geef a.u.b. hieronder een beschrijving:
- ☐ Anders:

Zijn er ook andere personen in uw omgeving die het geluid waarnemen (meerdere antwoordopties mogelijk)?

- ☐ Nee
- ☐ Ja
  - ☐ Partner
  - ☐ Familieleden
  - ☐ Huisgenoten
  - ☐ Buren
  - ☐ Bezoek
  - ☐ Collega's
  - ☐ Anders:

Hebt u onderzocht of bepaalde maatregelen uw hinder van het laag frequente geluid verminderen (meerdere antwoordopties mogelijk)?

☐ Nee

	Zo ja, hebben deze maatregelen uw hinder vermindert?	
☐ Indoen van oordopjes	☐ Ja	☐ Nee
☐ Sluiten of juist openen van ramen	☐ Ja	☐ Nee
☐ Hard(er) aanzetten van radio of televisie	☐ Ja	☐ Nee
☐ Verplaatsen van het bed	☐ Ja	☐ Nee
☐ Uitzetten van een verdachte bron	☐ Ja	☐ Nee
☐ Afzetten van stroom en accu/batterij/apparatuur	☐ Ja	☐ Nee
☐ Anders:	☐ Ja	☐ Nee

International Journal of
Environmental Research and Public Health

MDPI

Vragen met betrekking tot de ondervonden hinder

Hoe vaak ervaart u overlast door laag frequentgeluid?

- ☐ Nooit
- ☐ Soms
- ☐ Regelmatig
- ☐ Vaak
- ☐ Continu

Inwelkematevoeltuzichbeperktinuwalledaagseleven doorhetwaarnemen van laag frequentgeluid?

Helemaal niet (1)	1	2	3	4	5	6	7	8	9	10	Heel erg (10)

Zijn er klachten die u aan het waargenomen geluid wijt (meerdere antwoordopties mogelijk)?

1. Lichamelijke klachten (meerdere antwoordopties mogelijk)

- ☐ Drukkend of pulserend gevoel in/op de oren
- ☐ Druk op de borst
- ☐ Gevoel van trillingen in lichaam
- ☐ Duizeligheid
- ☐ Hoofdpijn
- ☐ Nekpijn of pijn in de rug
- ☐ Hartklachten (bijv. hartkloppingen)
- ☐ Kortademigheid of oppervlakkige ademhaling
- ☐ Anders:

International Journal of
Environmental Research and Public Health

MDPI

2. Psychologische klachten (meerdere antwoordopties mogelijk)

☐ Irritatie

☐ Prikkelbaarheid

☐ Concentratieproblemen

☐ Slaapproblemen

☐ Vermoeidheid

☐ Benauwdheid

☐ Stress

☐ Onrust

☐ Angst

☐ Somberheid

☐ Anders:

3. Sociaal maatschappelijke gevolgen (meerdere antwoordopties mogelijk)

☐ Baanverlies

☐ Arbeidsongeschiktheid

☐ Relatie/gezinsproblemen

☐ Huisvestingsproblemen

☐ Anders:

Hebt u melding van het laag frequentgeluid gemaakt bij

☐ Geen melding gemaakt

☐ GGD

☐ Gemeente

☐ Provinciale Omgevingsdienst

☐ Woningbouwvereniging

☐ RIVM (RijksInstituut voor Volksgezondheid enMilieu)

☐ Anders:

International Journal of
Environmental Research and Public Health

MDPI

Hebt u in verband met uw klacht(en) deskundigen geraadpleegd?

☐ Huisarts

☐ Keel, neus en oorarts

☐ Psycholoog

☐ Psychiater

☐ Audioloog

☐ Neuroloog

☐ Cardioloog

☐ Maatschappelijk werk

☐ Anders:

Wat was het resultaat van de raadpleging van deze deskundige? Beschrijf a.u.b. alle uitkomsten en/of diagnoses.

- ....
- ....
- ....
- ....
- ....
- ....
- ....
- ....

Bent u gediagnosticeerd met tinnitus (oorsuizen) door een deskundige?

☐ Ja

☐ Nee

☐ Nee, ik ben niet gediagnosticeerd met tinnitus, maar ik denk wel dat ik tinnitus heb.

Bent u gediagnosticeerd met een neurologische aandoening door een deskundige (bijv. hersentumor of hersenbloeding)?

☐ Nee

☐ Ja

Zo ja, waarmee bent u gediagnosticeerd?

Bent u gediagnosticeerd met een psychiatrische of psychologische aandoening (bijv. depressie, angststoornis, psychose of schizofrenie)?

☐ Nee

☐ Ja

Zo ja, waarmee bent u gediagnosticeerd?

Gebruikt u medicijnen? Noteer de gebruikte medicijnen hieronder.

**Medicatie**	**Dosis (b.v. mg)**	**Frequentie (b.v. 1x per dag)**
